# News analysis

**DOI:** 10.1136/tc.2009.032169

**Published:** 2009-07-17

## AUSTRALIA: MAKING INDUSTRY CONTACT TRANSPARENT

All articles written by David Simpson unless otherwise attributed. Ideas and items for News Analysis should be sent to: d.simpson@iath.org

Tobacco control advocates worldwide will recognise a particular scenario from the bad old days. After trying for months to meet the health minister to discuss tobacco control policy, using every available contact, perhaps marshalling a top level team of the great and the good from your country's medical profession, eventually, after false starts and postponements, you get a meeting, to find that the minister has been called away. You have been palmed off with an official, and a not very senior one, for an inconclusive meeting that advances your cause not one iota.

Then you find that tobacco industry executives had waltzed into the minister's office weeks ago, soon after demanding a meeting, or even had a working lunch with the minister and senior officials. They got a preview of forthcoming policy, the better to plan its circumvention; and you later find that other such meetings had taken place, with tobacco companies actually helping to shape the policy into what they, backed by their friend the finance minister, had agreed they could work with, meaning business could continue as usual, with uninterrupted recruitment of young people to smoking. Naturally, when this happened, you felt a unique sense of outrage. One of the fundamental injustices of tobacco control policy had been illustrated, and in a most distasteful way.

Now, however, guidelines on how to implement the Framework Convention on Tobacco Control (FCTC) can help make such absurd situations history, because they have been designed by experienced people and organisations genuinely concerned about health, not tobacco company profits. Article 5.3 of the FCTC says governments should protect their health policies from commercial and other vested interests of the tobacco industry; and one of the guidelines adopted by the conference of parties in November 2008 specifies that parties (governments), when dealing with the tobacco industry or those working to further its interests, should be accountable and transparent.

Among the first off the mark, as so often, is Australia, whose Department of Health and Ageing has begun notifying the wider world about its meetings with the industry. Its website says that on 22 January this year, for example, a meeting was held with British American Tobacco Australasia (BATA), which fielded its head of public affairs and director of corporate and regulatory affairs. Issues discussed included points in BATA's response to a recent government report, Tobacco Control in Australia: Making Smoking History; how article 5.3 guidelines will apply to the industry; and the timeframe relating to the availability of a government sponsored evaluation of graphic health warnings.

Australian colleagues are asking for further details of the meeting, as they will, no doubt, in respect of subsequent meetings. But even the existing detail is, in mathematical terms, an infinite improvement on past practice in all too many countries, and a commendable example of an important aspect of the FCTC and its guidelines being put into practice.

## JAPAN: AVOID SWINE 'FLU, HAVE A SMOKE

In Japan, especially in winter, it is not uncommon to see people going about their business in shops, offices and in the street wearing white face masks. Japan must be the undisputed world leader in politeness and the masks usually just mean that the wearers have coughs or colds, and do not want to pass any infections to their fellow citizens.

Earlier this year, as the world first began to hear about swine influenza, kiosks and pharmacies in Japan saw brisk and rising demand for special face masks marketed in response to the high level of citizen awareness of the possibly fatal results of catching the disease. While Japan has made much progress in recent years in trying to tackle another, far larger and longer established cause of premature death—smoking—health advocates know there is still a long way to go to educate the general public about the relative risks of smoking in the context of other potential causes of disease. So it was no surprise that the irony of displaying cigarettes in close proximity to anti-swine-'flu masks was apparently lost on some shopkeepers. The only surprise, said some observers, was that the manufacturers or retailers had not pre-drilled a small hole in each mask, to accommodate a reassuring cigarette.

**Figure:**
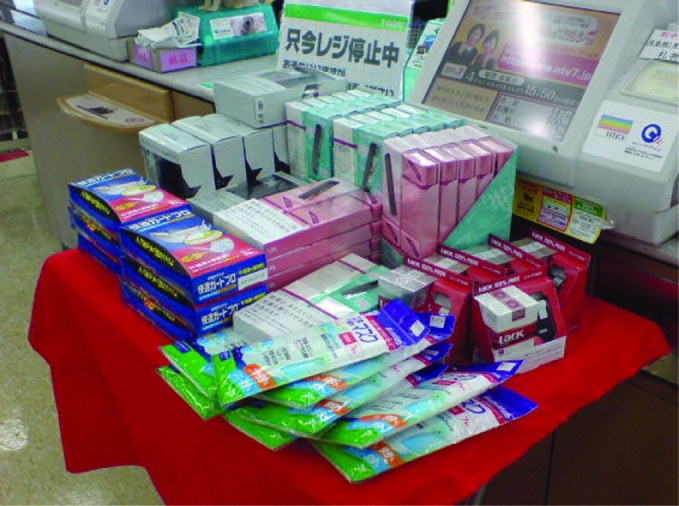
Japan: cigarette packs on display with another fast-selling, disease-related item of merchandise, anti-swine 'flu masks, at a convenience store earlier this year.

## WORLD: GRAPHIC WARNINGS WORK!

Article 11 of the World Health Organization's Framework Convention on Tobacco Control (FCTC) sets out minimum requirements for warnings on tobacco packs and related materials. These have been known, from the earliest days of tobacco control, almost certainly with the encouragement of tobacco companies, as health warnings. In reality, of course, they are disease warnings, concerning as they do some of the most lethal and unpleasant diseases from which a high proportion of tobacco users die prematurely.

A new report on the effectiveness of health warnings has been published by the International Tobacco Control (ITC) collaboration project. The collaboration is an ongoing international longitudinal study currently in 19 countries around the world, including both high and low income countries. Prepared for World No Tobacco Day on 31 May, the report is based on cross-sectional and longitudinal studies of text and pictorial warnings across many ITC countries. It concludes that graphic pictorial warnings are more effective than text-only warnings. Specifically, they are more noticeable and dominant than text warnings, heighten awareness about the harmfulness of smoking and motivate smokers to stop smoking.

The report's findings provide compelling evidence of the effectiveness of pictorial warnings and support the strong FCTC Article 11 Guidelines, adopted at the Third Conference of the Parties in November 2008, which call for pictorial warnings on at least 50% of the package. In addition to the wealth of information and illustrations in the report, interviews with key experts involved in research on health warnings are available online.

The report, entitled Evidence and Recommendations from the ITC Project FCTC Article 11—Tobacco Warning Labels, can be downloaded at: http://roswelltturc.org/ITCwarningreport.pdf and the audio interviews are at: http://roswelltturc.org/ .

**Figure:**
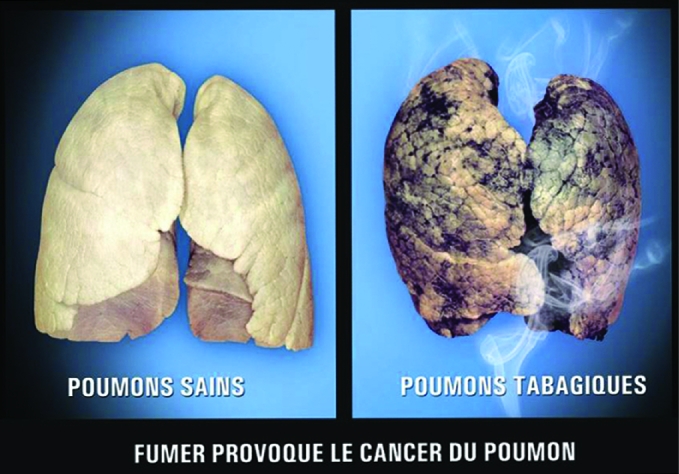
This lung cancer warning from Mauritius, showing a healthy lung beside a smoker's lung, is one of many illustrations in the new health warnings report.

## CANADA: BAT'S “GREENWASH”

Attempts by Imperial Tobacco Canada, the wholly owned Canadian subsidiary of British American Tobacco (BAT), to advertise its new “environmentally friendly” cigarette packaging were met with ridicule in May. The company was lambasted in *The Toronto Star*, in an article written by the newspaper’s environment reporter, after advertisements for its du Maurier cigarettes appeared in numerous publications across Canada. The ads, published in *Toronto Life* magazine, free entertainment weeklies and in bar bathrooms, said: “We have updated our packaging to reduce its impact on the environment.” They stated that Imperial had replaced foil wrapping with paper and its cardboard packaging now “meets standards supporting sustainable forest management.”

But commentators in the *Star* were not buying it. Gideon Forman of the Canadian Association of Physicians for the Environment laughed when asked about it. Later, when he stopped laughing, he said, “Is it green washing? Yes. Are they making a product that is still toxic to people’s health? Yes. So they are now making a product with a little bit more paper. Big deal.”

A comprehensive definition of “greenwashing” is provided by the *SourceWatch Encyclopedia*: greenwashing is “the unjustified appropriation of environmental virtue by a company… to create a pro-environmental image, sell a product or a policy, or to try and rehabilitate their standing with the public and decision makers after being embroiled in controversy.”

Imperial’s attempt at greenwashing may be related to a growing chorus of voices which are demanding action on cigarette butt litter. A recent newsletter by the David Suzuki Foundation, a well-known science-based Canadian environmental organisation, noted that, “From start to finish, cigarettes are bad for the environment. Tobacco production uses land that could support food crops or forests, and pesticides used on tobacco farms harm workers and pollute the soil and water. An incredible amount of fuel is needed to dry tobacco... At least 4.5 trillion non-biodegradable filter cigarette butts are discarded around the world every year.”

By trying to portray itself as a responsible corporate citizen environmentally, Imperial may be attempting to get “out front” on the issue, before the chorus of voices demanding action on tobacco-product-related litter becomes a roar which governments can no longer afford to ignore.

Progress on cigarette butt litter is found in both Edmonton and San Francisco *see this issue, page 255* where mayors there have recently said it would be worthwhile to levy a tax on tobacco products to help offset the clean up costs related to cigarette butts. Meanwhile, city officials in Ville Marie, a downtown borough in Montreal, have begun enforcing a two-year-old public cleanliness bylaw and are issuing C$169 (US$149) tickets to smokers who discard their cigarette butts on streets and sidewalks.

Attempts to give themselves a green sheen are not uncommon among tobacco companies. The industry has surely noticed an upswing in concern for the environment by citizens in Canada and around the world, largely due to scientific findings related to climate change. For example, annual reports to shareholders by Japan Tobacco (and other multi-nationals) have detailed the company’s efforts to reduce the greenhouse gas emissions associated with its operations.

Most people aren’t fooled, however, and can see through the green sheen. The headline in the *Star* article says it all: “Smokes: first you’ll feel green, and then you’ll die.”

### 

TREVOR HACHÉ

Non-Smokers’ Rights Association, Canada

thache@nsra-adnf.ca

**Figure:**
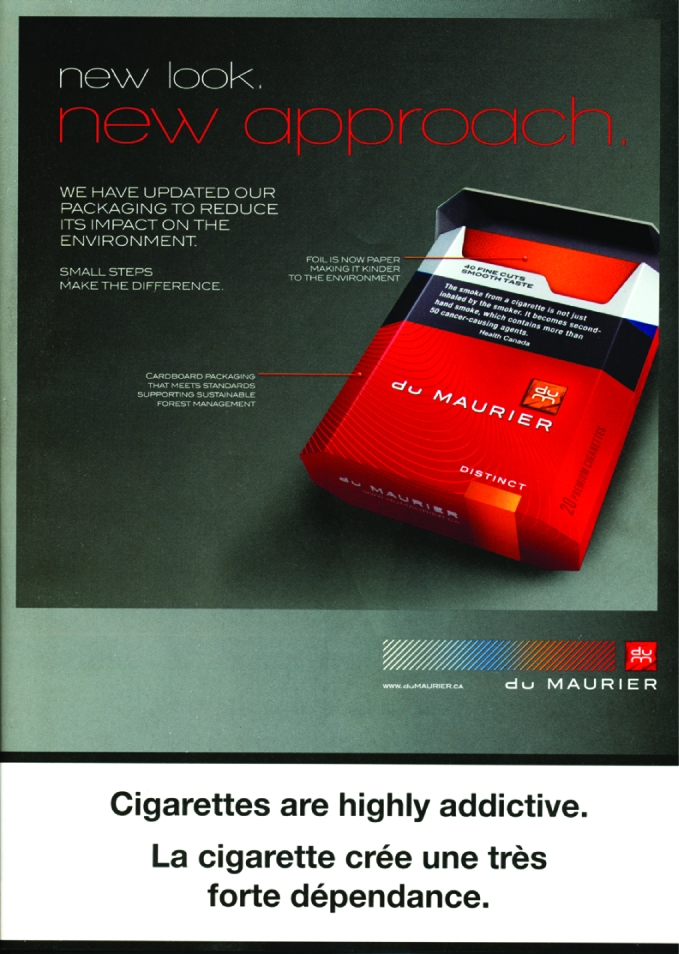
Canada: in this ad, published in the June 2009 edition of *Toronto Life* magazine, BAT's Canadian subsidiary, Imperial Tobacco Canada, indicates what it claims are environmentally friendly improvements to its du Maurier cigarette pack, while getting some promotion into the bargain.

## USA/WORLD: TOP CDC CHOICE APPLAUDED

Public health professionals in the United States and around the world have been praising President Obama's announcement in May that he has chosen Dr Thomas R Frieden, New York City's health commissioner, to be the next director of the Centers for Disease Control and Prevention—CDC. Dr Frieden is a public health doctor best known for his pioneering work to make New York a substantially smoke-free city, under the leadership of mayor Michael R Bloomberg. In addition, he has first hand experience of working in low income countries, which given CDC's international programmes and influence, cannot but be good news for global tobacco control.

Many also see this appointment as giving hope for a new beginning for US policy on tobacco. Despite ground-breaking progress by individual states, counties and cities, the USA has for decades evaded serious tobacco control leadership at the federal level. While the Bush administration spent more than $50 billion on anti-bioterrorism initiatives, for example, it seemed less concerned by the more than 400,000 annual deaths from tobacco. When appointed to the New York post shortly after the “9/11” terrorist attacks, and asked about challenges such as bioterrorism in contrast to his stated intention of prioritising anti-smoking initiatives, Dr Frieden replied, “Bioterrorists are not going to kill more New Yorkers than tobacco.”

## USA: NURSES QUIZ DIRECTORS

The first Philip Morris International (PMI) annual stockholders' meeting was held on 5 May at the Grand Hyatt Hotel in New York City. PMI is the largest tobacco company in the United States, manufacturer of Marlboro, the world's most successful cigarette brand, and owns seven of the world's largest 20 tobacco companies. The chairman and chief executive officer, Louis C Camilleri, noted in his opening remarks to shareholders that the key achievement of 2008 had been PMI's successful spin-off from Altria Inc, and that the company continued to be committed to high financial returns.

Analysts, however, note that the tobacco industry is being forced to wrestle with adverse global economic forces, including litigation and government regulation, increasingly imposed smoking restrictions, and health concerns related to the use of tobacco products and to exposure to environmental tobacco smoke. In other words, to keep up these high fiscal returns, tobacco companies need to find more smokers.

Activists from around the world once again came together at the shareholders' meeting to protest about youth being targeted by the tobacco industry as a developing market. I attended the meeting with two other nurses, as members of the Nightingales Nurses, a group that works to educate other nurses and the public about the role of the tobacco industry in creating disease. For six years we have challenged the tobacco industry by attending various tobacco company shareholder meetings and presenting the truth about the effects of the companies' products (see, *inter alia*, USA: nightingales sing at PM’s AGM. *Tob* *Control* 2004;**13**:218).

Before the meeting, we held signs and distributed information to passers-by during rush hour on one of New York’s busiest streets. We joined representatives from anti-smoking organisations and watchdog groups including Corporate Accountability International, Essential Action, the Campaign for Tobacco-Free Kids and Rebel for an international day of action. The New York City Police challenged us to keep away from the hotel entrance and shareholders “for our own safety.” Later, as we attended the meeting with proxy votes, we listened to Mr Camilleri’s remarks and waited for the question and answer period. Rules called for comments to be limited to two minutes and to avoid social commentary. During the one hour period three of those who approached the microphone congratulated the chief executive officer on his financial success, but the rest of the speakers chose to ask questions designed to show that PMI’s marketing tactics were not nearly as “socially responsible” as the company claimed.

Nancy Wise, Joan O’Connor and I wore white coats to distinguish ourselves as health professionals. We read statements from our patients and their families about the suffering they had endured, and asked questions designed to make the attending shareholders acknowledge that their profits came at a great human cost. After each question or comment, however, our microphones were cut off, prohibiting any follow-up, which was in itself a form of answer. When I asked if PMI had ever paid a fine related to marketing to youth and if there was any pending litigation concerning this, Mr Camilleri responded that PMI had never paid a fine. We were unable to get any specific details about litigation.

Jonathan Romo, an activist from Mexico, asked why PMI sponsored rock concerts if its advertising intent was to get established smokers to change brands as it claimed, not to entice young people to start smoking. Camilleri responded that PMI abided by Mexican law and that the Mexican Federal Commission of Protection Against Health Risks sent two representatives to the concert to ensure compliance. However, he had noted in his opening remarks that one of PMI’s directors, Carlos Slim Helu, was unable to attend the annual meeting because of the “current situation in Mexico,” referring to the swine 'flu outbreak and the consequent limits placed on Mexican travellers.

In her statement, Rachel Kitonyo from Kenya asked Camilleri to stop selling cigarettes to African children where the governments are already overwhelmed dealing with HIV/AIDS, tuberculosis and malaria. Camilleri’s direct response was that “we don’t market to children” and that in 2008 PMI sold less than 50 million cigarettes in all of East Africa, none in Nigeria for at least a decade and none in Kenya. However, a photo of a 2005 Marlboro billboard in Nigeria would dispute his answer. The CEO ended his response to Kitonyo by thanking her, with what could only be taken as sarcasm, for “coming all the way from Nairobi.”

For many present, the best question of the day came from Ashley Herrin, of the US group Students Working Against Tobacco. She prefaced it by saying she realised that no matter what she asked, Camilleri would probably tell her, as he had previous questioners, that she was ill-informed or had misunderstood the facts. So instead of a factual question, she asked for “a minute of silence on behalf of the 5.4 million people who die from tobacco-related illness each year.” Camilleri looked to his right for advice from his associate, who nodded assent. The audience seemed surprised as the room fell into complete silence. It seemed like a very long New York minute before the chief executive took the next question.

Finally, a short “feel-good” movie about the Indonesian tobacco company Sampoerna was shown. It is estimated that seventy per cent of Indonesian men smoke, but the movie showed mostly women working at the company. Husbands and children of the female employees expressed gratitude for the benefits extended to the families, allowing them to send their children to school or begin business ventures of their own.

Tight security at the entrance to the meeting failed to stop six protesters who stood with veils over their faces and raised signs showing a wolf in sheep’s clothing to the shareholders and directors attending.

We were a small group facing well-oiled Big Tobacco, but the protest was given critical validation in the Business Section of the *New York Times* the next day, 6 May 2009, which noted that there were “30 dissidents” in the crowd of 200. Despite the annual presence of international protesters, never before had they gained coverage in the mainstream business press. Thirty may not be a large number, to be sure, but this 30 may possibly have been enough to give executives and shareholders of Philip Morris International pause for thought.

### 

CHRISTINE CONTILLO

New Jersey, USA

nursemomnj@aol.com

## PAKISTAN: BAT SPONSORS CLIMATE CHANGE EVENT

Health advocates in Pakistan were shocked and disappointed by their government's acceptance of British American Tobacco's Pakistan subsidiary, Pakistan Tobacco Company (PTC), as co-sponsor of a recent government-backed climate change event. The ministry of the environment apparently saw no incongruity in associating an industry whose products, more than any other sector's, destroy that most essential part of the environment for smokers, the human body, with organised concern about the external environment. Perhaps the ministry was lulled into a false sense of normality by the participation of the university of Manchester, UK and the British government itself, through its High Commission in Pakistan. Action on Smoking and Health UK has requested explanations.

**Figure:**
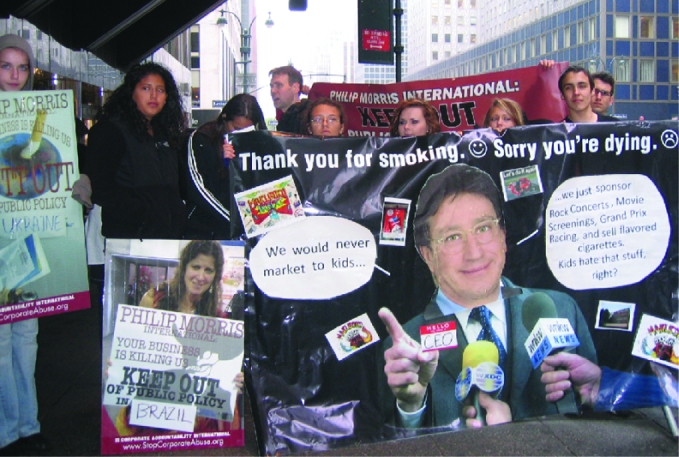
USA: nurses and other protestors outside the annual general meeting of Philip Morris International, in New York City.

## NEPAL: NEWSPAPER'S SPECIAL EDITION

On the occasion of World No Tobacco Day (WNTD) on 31 May, a weekly national language newspaper published a WNTD special issue focused on tobacco health warnings, emphasising the message, “Show the truth, picture warnings save lives.” The name of the newspaper, *Amalekh*, means liberty from various forms of slavery or dependence in society. Launched in 2006, it is the country's first tobacco-free newspaper. The special issue, the first to mark WNTD in Nepal, included editorials on tobacco free culture, smoking in Nepal, the history of tobacco, the impact of health warnings around the world, with pictures of actual warnings and a calendar for 2009, as well as interviews with local tobacco control leaders and a case history of a young lung cancer patient who died last year aged just 34. It also contained information about diseases that are caused by smoking, and tips on how to give up.

## USA: SMOKERS TO PAY FOR LITTER?

The mayor of San Francisco, California, has proposed that an additional state tax should be added to the price of cigarettes to pay for the litter that smokers leave in the city's streets. If passed, it would be the first such scheme in the United States, where cigarette waste, especially filters, which are not biodegradable, constitutes a major environmental problem, accounting for a quarter of all street litter. Initial estimates suggest that the new tax, to cover cleaning costs, may be around 30 US cents per pack.

## PAKISTAN: WHO HONOURS DR KHAN

The World Health Organization has awarded a World No Tobacco Day medal to Dr Javaid Khan, a leading chest physician and tireless campaigner over many years for the much tougher tobacco control measures that his country so badly needs. Despite heavy teaching duties as professor and head of pulmonary and critical care medicine at the Aga Khan university in Karachi, and onerous clinical work, Dr Khan has somehow found the time to be a human whirlwind of tobacco control activity. He has monitored the tobacco industry's activities, raised tobacco issues at countless medical meetings, pressed his government for a tougher, less industry-friendly policy on tobacco, and taken part in countless media activities. All this work has been motivated by seeing such frequent examples of Pakistan's massive burden of tobacco induced disease. But as he told this journal recently, “Each time I see a case of lung cancer or chronic obstructive pulmonary disease, I somehow get extra strength to do more in tobacco control.” Among the best evaluations of his work have been attempts by tobacco companies to hold “stakeholder” discussions with him, which he has declined.

In addition, Dr Khan has repeatedly publicised the rise of youth smoking, fanned by relentless targeting of young people by a seemingly ever more subtle range of promotional techniques. He is chairman of the national alliance for tobacco control, a model coalition of key organisations and individuals. When the occasion has demanded it, he has thought nothing of taking to the streets with colleagues, all equipped with appropriate placards, to get across their message in a way that would have been unthinkable when tobacco control was first getting under way in Pakistan just two decades ago.

**Figure:**
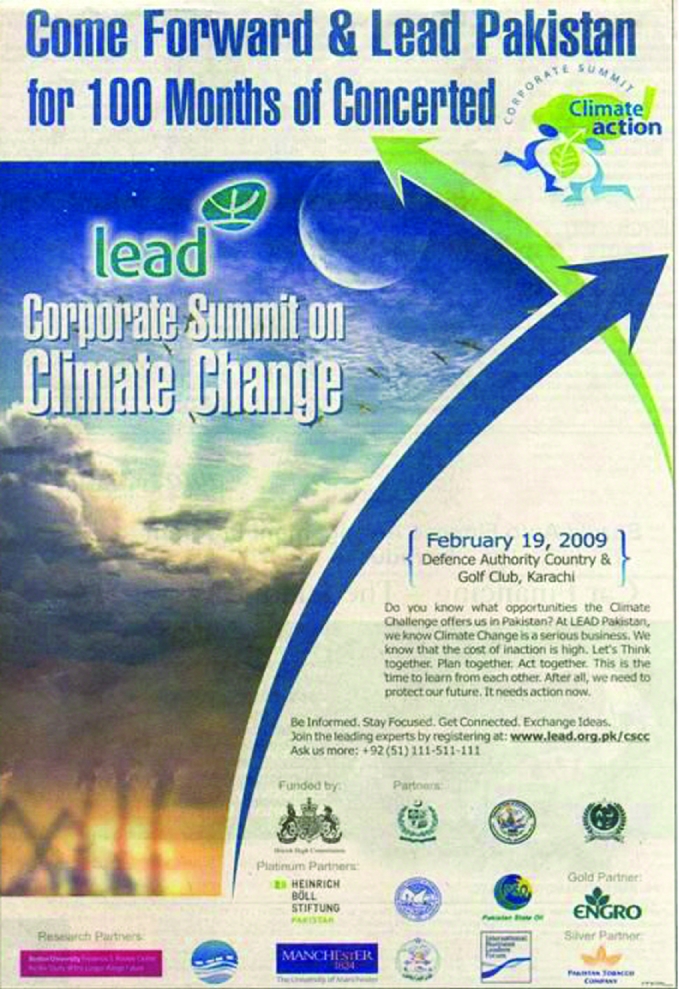
Pakistan: a poster advertising a climate change event, bearing the logo of BAT's Pakistan subsidiary in the lower right hand corner.

**Figure:**
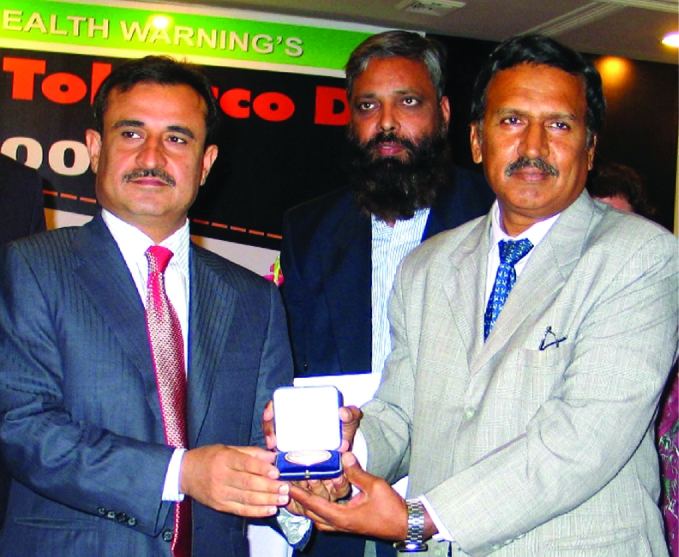
Pakistan: Dr Javaid Khan (right) receives his WHO medal from Pakistan's health minister, Mir Aijaz Hussain Jakhrani, watched by WHO's tobacco programme officer in Pakistan, Mr Shahzad Alam Khan.

